# Focal seizures unfold variably over time

**DOI:** 10.1093/braincomms/fcad230

**Published:** 2023-08-25

**Authors:** Maxime O Baud, Vikram R Rao

**Affiliations:** Sleep-Wake-Epilepsy Center, Center for experimental Neurology, NeuroTec, Department of Neurology, Inselspital Bern, University Hospital, University of Bern, Bern 3010, Switzerland; Department of Neurology and Weill Institute for Neurosciences, University of California, San Francisco 94143, CA, USA

## Abstract

This scientific commentary refers to ‘Chronic intracranial EEG recordings and interictal spike rate reveal multiscale temporal modulations in seizure states’ by Schroeder *et al*. (https://doi.org/10.1093/braincomms/fcad205).

**This scientific commentary refers to ‘Chronic intracranial EEG recordings and interictal spike rate reveal multiscale temporal modulations in seizure states’ by Schroeder *et al*. (https://doi.org/10.1093/braincomms/fcad205).**

A central dogma in clinical epileptology holds that seizures are random in their time of occurrence but stereotyped in the symptoms they produce. Both features have relevance for people living with epilepsy: seizure unpredictability is a major source of disability,^1^ and stereotypy helps distinguish epileptic seizures from other paroxysmal neurological events. Yet, clinicians have long had clues that canonical notions of randomness and stereotypy belie the complexity of seizures. For example, observations of cyclical patterns in seizure occurrence date back to antiquity,^1^ and electroclinical manifestations can vary from one seizure to the next (Fig. 1). Indeed, fundamental tenets in epilepsy have been challenged recently by growing evidence for an underlying temporal structure that both explains seizure timing and creates seizure variability.

**Figure 1:**
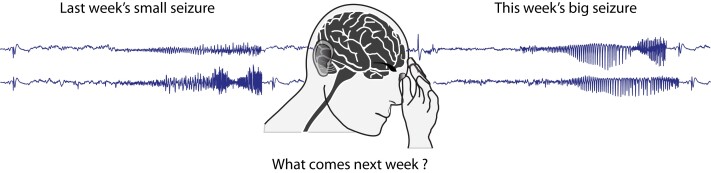
**Time-varying focal seizures.** Electrographic seizures and their accompanying symptoms may vary from one occurrence to another.

A contemporary, probabilistic conception of seizure timing posits the existence of alternating high and low states of seizure likelihood, defined in relation to cyclical fluctuations in epileptic brain activity.^2–7^ The availability of chronic intracranial EEG recordings over years in some people with focal epilepsy has revealed that interictal epileptiform activity (IEA) fluctuates periodically over multiple timescales, from circadian (∼24 h) to longer cycles of about-weekly to about-monthly duration, so-called multidien (multi-day) cycles.^2,3,5^ Seizures preferentially occur at certain times during each cycle, most likely when critical phases of co-existing circadian and multidien cycles align.^3^ Whether seizures occurring at different phases may have phase-specific characteristics has remained an open question. Conceivably, the ‘seizure pathway’—the spatiotemporal dynamics with which a seizure unfolds—may also demonstrate temporal variability. In other words, do cycles of IEA help determine *how* a seizure manifests in addition to *when* it occurs? Does the *type* of seizure depend on the *time* of the seizure? The answers to these questions have relevance for the network theory of epilepsy^8^ but also implications that bear directly on clinical practice. For example, to the extent that electrographic patterns relate to salient semiology, such as duration, loss of awareness, and convulsions, methods to predict the clinical manifestations of seizures could enable tailored, time-varying therapies. The discovery of cycles in epilepsy has made seizure forecasting a near-term reality,^1^ but the ability to anticipate nuanced features of seizures would help align the intensity of preventative interventions with the expected severity of seizures.

Recently in *Brain Communications*, Schroeder and colleagues^9^ addressed some of these questions by analysing long-term human intracranial recordings from the NeuroVista trial, a unique dataset that has yielded fundamental insights on seizure dynamics.^2,4^ Despite its modest sample size (*N* = 10 participants were analysed for this study), strengths of this dataset include its chronicity (185–767 days of recording time) and a large number of events (57–452 seizures/participant), which enable analyses of seizure variability within individuals. Using a soft-clustering technique called non-negative matrix factorization, applied here on six frequency bands (delta to high-gamma), the authors deconstructed seizures into sequences of a finite number of seizure network states (SNSs). Thus, every seizure is expressed as an ordered progression of some or all SNSs, each with a duration that can be quantified and a time that can be related to momentary phases of circadian and multidien IEA cycles.

Seizures in the 10 participants were reduced to sequences of three to seven SNSs. Although focal seizures within an individual tended to start with the same SNS, the occurrence across seizures of subsequent SNSs was more variable in sequence and duration (up to 20-fold variation). As expected, all participants had circadian cycles of IEA (spike rate).^2,3^ In addition, six participants showed circadian modulation in the occurrence or duration of at least one SNS, showing that seizure characteristics can systematically vary according to time of day. Eight of the 10 participants had multidien cycles of IEA, with periodicities reminiscent of those found in a distinct dataset:^3,5^ 6 days, 10–15 days, and 28–29 days. Multidien IEA cycle phase modulated the occurrence or duration of specific SNSs in five participants, showing that seizure pathways can have cyclical variation over days to weeks. These effects were quantified using circular statistics, including the phase-locking value (occurrence of a SNS, discrete variable) and rank linear-circular correlations (duration of a SNS, continuous variable), tested against null distributions drawn from surrogate time-series. In this statistically robust approach,^10^ chance-level values are obtained by randomly shuffling the time of occurrence of seizures to quantify chance-alignments with the original underlying cycle. Over years of recording, new SNSs can appear (here, in 8/10 participants), suggesting that pathological networks underlying focal seizures may evolve at very long time-scales.^11^

Overall, these results can be distilled to two main take-home points: (i) seizures within individuals are stereotyped to the extent that they combine a set of SNSs, the prevalence and duration of which may progressively increase or decrease over time; and (ii) in some individuals, this stereotypy is modulated by certain phases of circadian and multidien IEA cycles. Taken together, these findings advance our understanding of seizure variability by revealing cyclical dynamics in seizure pathways.

This study has some limitations, which are acknowledged by the authors, including the small sample size and limited spatial coverage of intracranial electrodes. Unfortunately, the SNS composition of seizures was not directly relatable to seizure severity or clinical semiology, such as the occurrence of secondary generalization, a principal determinant of seizure-related morbidity. Hence, the clinical utility of knowing when certain SNSs are more or less likely to occur remains theoretical. Finally, although this study does well to highlight the existence and multidimensionality of seizure variability, the results do not fully explain this variability. To this end, the authors propose several other features that could be analysed in future work, including high-frequency oscillations and other patterns of neural activity besides spikes.

Like much work in the field, this study focuses on seizures, the most obvious expression of epilepsy. Yet, the fact that cyclical patterns of activity in the interictal state correlate with both seizure timing and seizure pathways suggests that seizures may be co-modulated by factors that influence interictal network fluctuations. Seizures are increasingly understood as expressions of dynamic epileptic networks as opposed to chaotic perturbations of unwavering ‘normal’ activity.^8^ As such, it will be critical for future studies to dissect network behaviour in the interictal state by identifying drivers of cyclical phenomena and by characterizing the effects of relevant clinical variables, like anti-seizure medications. For now, the elegant work of Schroeder *et al*. provides considerable inspiration for the work that lies ahead.
